# Efficacy of a smart glass-enhanced training programme for core doctor-patient communication skills among radiology residents in China

**DOI:** 10.1186/s41747-025-00630-w

**Published:** 2025-09-19

**Authors:** Yubin Xiao, Gengpeng Lian, Jiong Zhang, Qiafeng Chen, Huanpeng Wang, Lipeng Huang, Hongwu Yang, Chunmin Zhu, Wei Mei, Caiyu Zhuang, Chaosen Zhong, Ruibin Huang

**Affiliations:** Department of Radiology, The First Affiliated Hospital, Shantou University Medical College, Shantou, China

**Keywords:** Educational technology, Internship and residency, Patient satisfaction, Simulation training, Smart glasses

## Abstract

**Background:**

Effective doctor-patient communication (DPC) skills are critical competencies in residency training. This study evaluated the efficacy of a smart glass (SG)-based communication skills training curriculum for radiology residents in China.

**Materials and methods:**

This quasi-experimental study with a one-group pretest-posttest design involved 18 radiology residents in an 8-week SG-based DPC simulation training. Supervisors used the SEGUE scale, while residents and four standardised patients (SPs) trained by the Guangdong Institute of Simulation Medicine self-assessed satisfaction with a Likert scale. Analysis compared pre- and post-training scores (before, immediately after, and 6 months post-programme). Post-training SG experiences were assessed via surveys.

**Results:**

Significant improvements were observed in SEGUE scale scores immediately and 6 months post-programme compared with pre-programme scores (17.06 ± 3.67 and 17.72 ± 3.12 *versus* 10.94 ± 2.88, respectively, *p* < 0.001). Similarly, Likert scores for SPs and residents showed significant increases both immediately and 6 months post-programme compared with initial scores (3.50 ± 0.51 and 3.67 ± 0.68 *versus* 2.39 ± 0.61, *p* < 0.001 for both, and 3.28 ± 0.46 and 3.55 ± 0.78 *versus* 2.66 ± 0.84, *p* = 0.037 and 0.008, respectively). Post-training, the Likert consistency between SPs and residents was 0.73 (*p* = 0.005). Of 18 participants, 16 (89%) reported that SG provided useful feedback, and 16 (89%) recognised the value of SG in developing DPC skills.

**Conclusion:**

The SG-based simulation training programme significantly enhanced and sustained DPC skills among radiology residents.

**Relevance statement:**

Smart glasses provide an innovative tool for recording standardised patient encounters, offering a perspective for analysing and evaluating residents’ interpersonal communication skills and nonverbal behaviours.

**Key Points:**

- Smart glasses enhance doctor-patient communication skills in radiology residents.
- Simulation training with smart glasses showed improvement in skills.
- Smart glasses offer a perspective for standardised patient encounters.
- They facilitate better analysis of residents’ interpersonal and nonverbal communication.

**Graphical Abstract:**

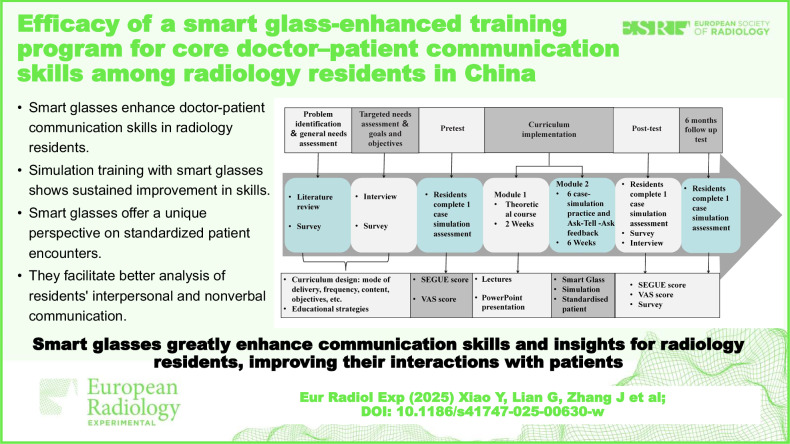

## Background

Doctor–patient communication (DPC) skills constitute one of the six core competencies in residency training and medical practice [1]. Effective communication with patients and their families or caregivers necessitates empathy, recognition of concerns, and engagement in decision-making processes [2]. However, in the 3-year radiology residency training programme in China, analysis of the 24 defined competency tasks reveals that the top 10 prioritised areas predominantly focus on patient care and medical knowledge domains. While this emphasis ensures strong technical training, it has resulted in the relative neglect of other critical competencies, especially essential “soft” skills such as interpersonal communication and patient interaction. This imbalance is further compounded by significant heterogeneity in training implementation across different regions and institutions nationwide, creating disparities in resident preparedness [3].

Radiology is integral to the patient care pathway and significantly influences the patient experience [2]. The Diagnostic Radiology Milestones, established by the Accreditation Council for Graduate Medical Education (ACGME) and the American Board of Radiology, stipulate that residents must proficiently convey “complex and difficult information, such as errors, complications, adverse events, and unfavourable news” [4]. As the field of radiology progresses towards a more patient-centred model, the significance of robust DPC skills is set to increase [5]. Recent studies indicate a preference among patients for direct discussions with imaging specialists regarding their imaging results [6–9]. Moreover, previous studies have highlighted the patient care benefits of such interactions, including decreased error rates, enhanced adherence to radiological recommendations, minimised delays in patient care, increased patient satisfaction, and reduced healthcare costs. However, the development of DPC skills among radiology residents typically occurs through on-the-job training or traditional didactic methods, offering limited opportunities for trainees to observe, actively engage, or reflect during the learning process.

Communication in medical encounters comprises both verbal and nonverbal elements [10]. It has been reported that nonverbal cues often dominate over verbal messages [11, 12]. Nonverbal behaviours, such as eye contact, posture, and tone of voice by physicians, substantially affect patient disclosure and satisfaction [11]. Video-based simulation assessments have demonstrated that reviewing and analysing one’s performance is crucial for ongoing professional development [13, 14]. However, traditional static cameras, used for recording videos for faculty and resident feedback and evaluation, capture only a limited range of key nonverbal communication behaviours during clinical encounters [15]. Recently, the advent of hands-off, wearable, interactive technologies such as smart glasses (SG) has prompted further research into their potential role in clinical education [16–18]. To date, our review has identified only a scant number of radiology residency programmes that provide formal training and evaluation programmes for DPC skills, with minimal evidence of their use in fostering the development of such skills in radiology residents [3, 19].

The aims of this study were: (1) to describe and evaluate the efficacy of a simulation-based DPC skills training curriculum for radiology residents, and (2) to provide proof of concept for the implementation of a first-person-view SG video recording method for procedural training and assessment.

## Materials and methods

### Study design and participants

This is a monocentric study conducted in the First Affiliated Hospital of Shantou University Medical College. The investigation was designed as a pre-post-intervention study, which commenced in May 2023. We recruited 18 radiology residents in the second or third postgraduate year to participate in the simulation programme. Participants were assessed using a scale to compare scores before and after the programme (pre-programme, immediately post-programme, and 6 months post-programme). The training was conducted over a period of 8 weeks.

### Programme curriculum

**Fig. 1:**
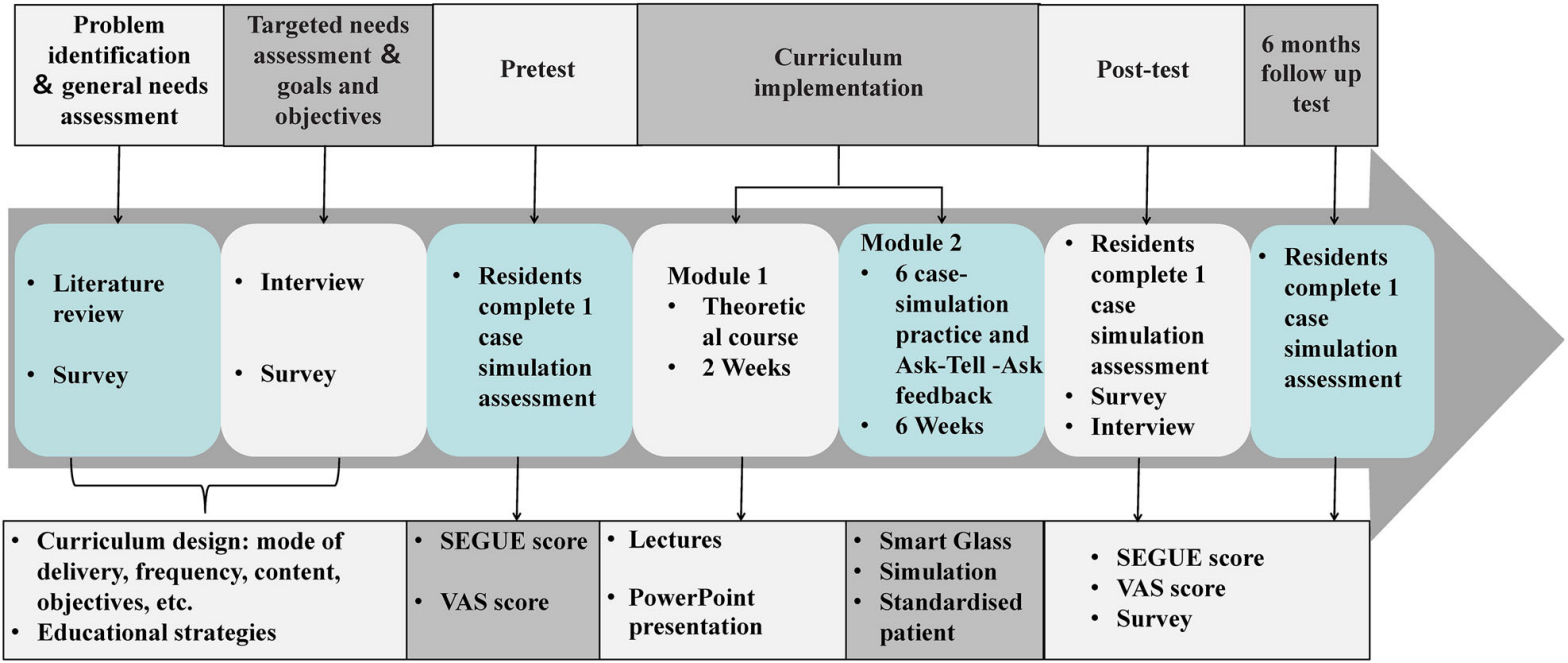
Schematic representation of the study design and procedures

The development of the DPC training programme was guided by an extensive literature review, expert seminars, and a thorough survey of resident needs. The results of the survey of training needs prior to the course can be found in our previous research work [20]. The curriculum comprised two modules, each designed to enhance progressively complex skills, ensuring that participants could effectively use DPC skills in both routine and challenging patient encounters (Fig. 1). The approach to the programme was learner-centred, competency-focused, and practical. Eighteen radiology residents were enrolled and divided into four groups. Each discussion group was supervised by an individual experienced in conducting clinical scenario simulations, employing small-group teaching methods, and utilising advanced communication techniques. Supervisors actively participated and demonstrated key skills during each module. The programme utilised four certified standardised patients (SPs), trained by the Guangdong Institute of Simulation Medicine to perform realistically in scenarios. These SPs underwent further training with a radiology faculty member skilled in communication and an acting coach to enhance the realism of the simulations.

The programme was structured into three main components: a 3-h module focused on theoretical knowledge, divided into three lectures aimed at developing well-rounded professionals who can communicate compassionately and effectively in diverse healthcare settings (Supplementary Table 1); an 18-h module emphasising practical simulation role-playing based on scenario guidance; and a 3-h assessment session, conducted three times—before the programme, immediately after, and 6 months later. Each assessment session lasted 1 h and evaluated the programme’s efficacy using a validated rating scale administered to SPs, radiology residents, and supervisors (Fig. 1). Drawing on the Calgary–Cambridge Guide and the realities of clinical practice in China [21–23], the curriculum integrated six common radiology communication challenges, namely error/apology, delivering bad news, cancelling an examination, challenging radiological reports, radiation risk counselling, and interactions with disgruntled referring physicians (Table 1). Each scenario was structured to include background information, role-play enactments, and specific instructions for the SPs.

**Table 1:** Contents of Module 2

Theme	Content	Duration
*Highlights*	• Skills learned through incorporating smart glasses into 6 cases of simulation-based practice and feedback. • Steps: supporting knowledge (0.5 h) → scenario simulation (1 h) → video review (0.5 h) → “Ask-Tell-Ask” feedback (1 h). • The presentation in each module is modified to the trainee’s needs and style based on personal experiences.	18 h
*Scenario* ***#1****: delivering bad news*	• Teaching purpose: breaking bad news. • Skills: create a private environment by closing doors, not interrupting, providing chairs for all, telling the truth empathetically (“I very am sorry, but the results are not in line with our expectations.”); encourage relatives to help, offering emotional or cultural assistance (“Is there anyone else who you would like to get involved in this?” “Do you have any spiritual, religious or other convictions that help you in challenging situations?”); offer hope to the patient (“There are a number of effective treatment options available for this condition”).	3 h
*Scenario* ***#2****: radiation risk counselling*	• Teaching purpose: to reassure young female patient and reduce anxiety, tension and discomfort while a male radiologist performs an air enema and explain radiation risk. • Skills: find out what the patient knows and thinks, explain effectively, make sure the patient understands the problem; gain the support of family members and suggest spiritual support for the patient.	3 h
*Scenario* ***#3****: error/apology*	• Teaching purpose: to interpret misdiagnosed radiology reports. • Skills: to elicit an explanation of the problem from the patient, explain the problem to the patient, and ensure the patient’s understanding.	3 h
*Scenario* ***#4****: Challenging radiological reports*	• Teaching purpose: interpret discrepancies in pre- and post-radiology reports. • Skills: to obtain an explanation from the patient of the problem, to explain the problem to the patient and ensure understanding; to explain the cause of these discrepancies, the possible course of the disease and the treatment and surgical procedures planned or underway.	3 h
*Scenario* ***#5****: angry referring physicians*	• Teaching purpose: communicate and interpret effectively with angry referral physician. • Skills: to elicit an explanation of the problem from the referring physician, explain the problem to the referring physician, and ensure the referring physician’s understanding.	3 h
*Scenario* ***#6****: cancelling examination*	• Teaching purpose: communicate and interpret MRI scanning contraindications. • Skills: to ensure understanding of the problem by finding out what the patient knows and thinks, and effectively educating and informing the patient in a popularisation style.	3 h

*MRI* Magnetic resonance imaging

**Fig. 2:**
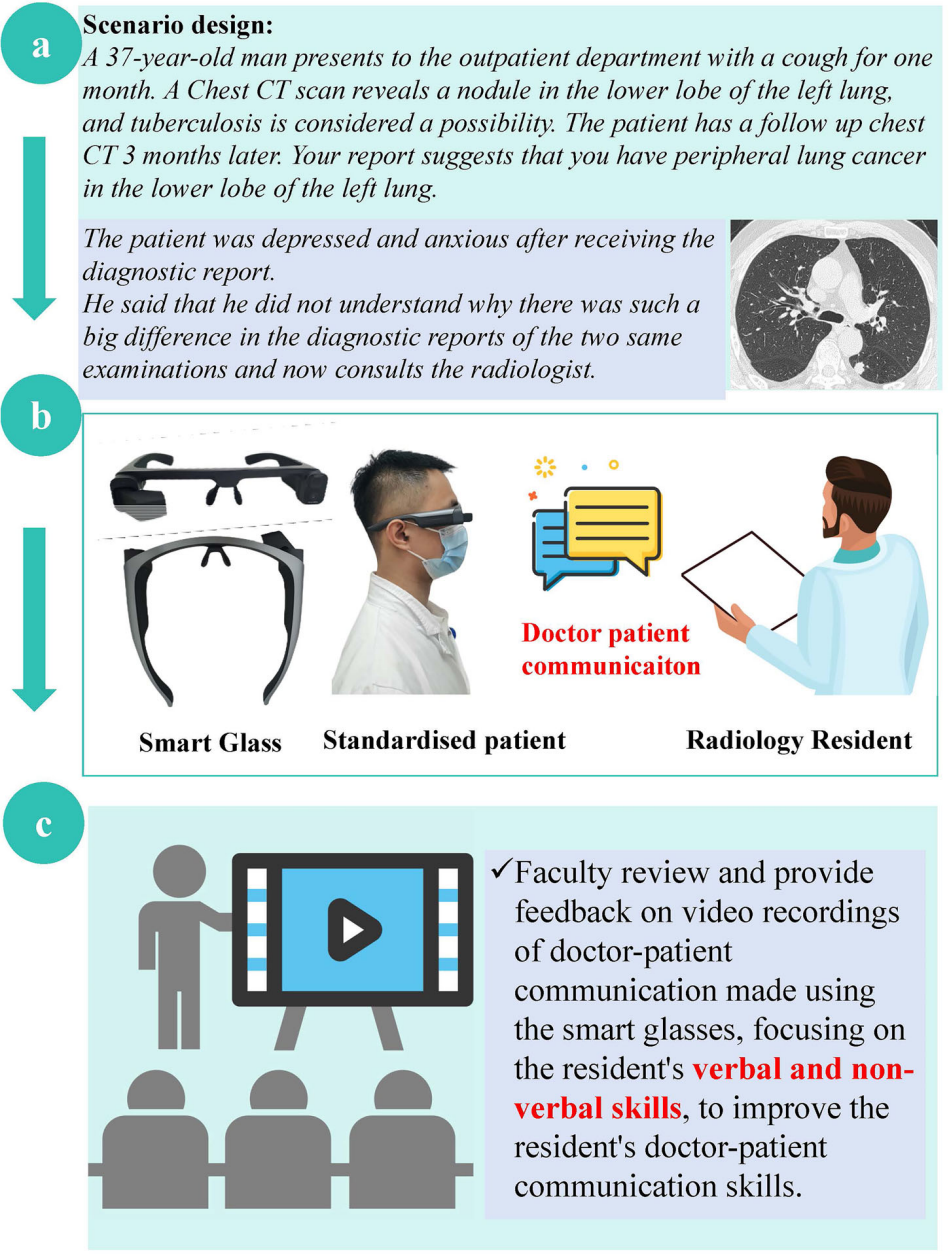
**a**–**c** Example of a simulation scenario showing a standardised patient wearing smart glasses during encounters and a debriefing session following the simulation

The format of the practical phase during scenario simulations and the subsequent debriefing sessions was structured as follows (Fig. 2):

- Step A: Prior to each simulation session, SPs received a standardised script for each topic, followed by 1 h of self-directed training using an SG (Eyeglass Camera, MJSV01FC; the user manual was provided in the Supplementary Table 4). Residents had 5 min to familiarise themselves with the scenario details on a computer outside the simulation room before engaging in the simulated scenario. These scenarios reflected realistic situations encountered by radiologists and patients, emphasising DPC in clinical contexts.
- Step B: During the enactments, SPs wore SG, which are wearable video recording devices, to capture first-person perspectives of the scenarios. SPs activated the SG recording before initiating the interaction, documenting their viewpoint throughout the encounter.
- Step C: Post-interaction, the SPs stopped the SG recording and transferred the SG to the technical support team for video download. Concurrently, SPs completed a checklist based on direct observation of the resident’s performance. The downloaded videos were set to start playback at a moment that highlighted a challenging question posed by the SPs, serving as a cue during the encounter. The residents then viewed these cued videos and received feedback on their verbal and nonverbal communication skills from the SPs and the supervising faculty during the debriefing session.

### Outcome assessment

The supervisors employed the SEGUE scale [24, 25] to evaluate the competence of residents following their consultations with the SPs. The SEGUE Framework consists of five dimensions and 25 items, each rated on a scale from 0 to 1, resulting in a composite score out of 25. The framework evaluates various aspects of the consultation process: ‘setting the stage’ (5 items), ‘eliciting information’ (10 items), ‘giving information’ (4 items), ‘understanding the patient’s perspective’ (4 items), and ‘ending the encounter’ (2 items). Post-encounter, both residents and SPs provided satisfaction ratings using a five-point Likert scale, ranging from 0 (extremely dissatisfied) to 5 (extremely satisfied), prompted by the statement: ‘Regarding the visit you just had with the patient, please indicate the score’. Assessments using the SEGUE and Likert scale were performed before, immediately after, and 6 months following the 24-h training programme. The post-session survey to assess attitudes towards the use of SG consisted of 10 dichotomous questions, reported as percentage responses (see details in Supplementary Table 2).

### Statistical analysis

Quantitative analysis of the data was conducted using SPSS (version 26.0; IBM Corp). Descriptive statistics, including mean, standard deviation, frequency, and percentage, were calculated for demographic data, observational data, and responses from the learning satisfaction survey. The means of repeated continuous variables were analysed using the Friedman test. A two-way mixed effects model estimated the intraclass correlation coefficients to assess interrater reliability [26]. A *p*-value of less than 0.05 (two-tailed with Bonferroni adjustment) was considered statistically significant.

## Results

A total of 18 radiology residents participated in this study. The mean age of the participants was 26.9 ± 1.9 years, and 50% (9/18) were female. Half of the residents were in their second year of residency, while the remainder were in their third year. All participants were included in each analysis, with no attrition noted between the pre- and post-intervention assessments.

**Fig. 3:**
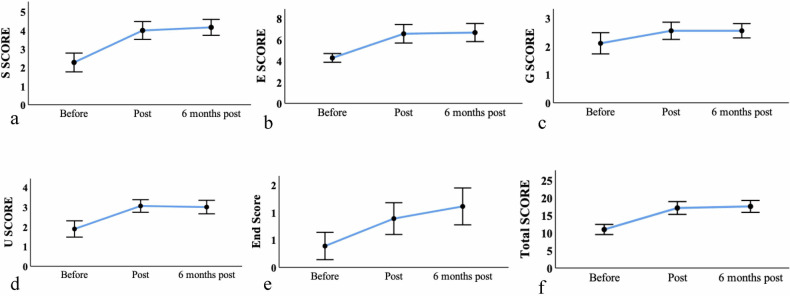
**a**–**f** Detailed score changes of the radiology residents as evaluated by the SEGUE scale before and after the programme. S, Set the stage; E, Elicit information; G, Give information; U, Understand the patient’s perspective; End, End the encounter

The mean SEGUE scale scores were 10.9 ± 2.9, 17.1 ± 3.7, and 17.7 ± 3.1 at pre-programme, immediate post-programme, and 6 months post-programme, respectively, indicating a significant increase from the pre-programme scores (*p* < 0.001). Most dimensions of the SEGUE scale exhibited statistically significant increases (*p* < 0.001) (Table 2, Fig. 3). However, no significant improvement was observed in the information-giving dimension, both immediately post-programme and 6 months post-programme (*p* = 0.182 and *p* = 0.157, respectively). Intraclass correlation coefficients between SPs and radiology residents were recorded as 0.53 (95% CI: -0.244 to 0.826, *p* = 0.062), 0.73 (0.267–0.897, *p* = 0.005), and -0.156 (-2.09 to 0.568, *p* = 0.616) at pre-programme, immediate post-programme, and 6 months post-programme, respectively. No differences were observed in SEGUE scores when analysed by gender at any time points (*p* ≥ 0.242) except in the “Understand the patient’s perspective” domain at 6 months post-programme. Compared with second-year residents, third-year residents scored significantly higher on the SEGUE scale both immediately and 6 months post-programme (*p* ≤ 0.011) in terms of “SEGUE total scores” as well as in the domains of “Set the stage” and “Elicit information” (see details in Supplementary Table 3). No statistically significant difference in satisfaction scores was observed between second- and third-year residents (median [interquartile range]: 4.0 [3.5, 5.0] *versus* 4.0 [3.0, 4.0]; Z = -0.960, *p* = 0.387).

**Table 2:** Data from the SEGUE and Likert scales before and after completion of the programme

Variable	Before the programme	Immediately after the programme	Six months after the programme	F value	*p-* value	Before *versus* immediately after the programme	Before *versus* 6 months after the programme
SEGUE total score	10.94 ± 2.88	17.06 ± 3.67	17.72 ± 3.12	30.68	< 0.001	< 0.001	< 0.001
Set the stage	2.28 ± 1.01	4.00 ± 0.97	4.17 ± 0.86	29.32	< 0.001	< 0.001	< 0.001
Elicit information	4.28 ± 0.83	6.55 ± 1.75	6.67 ± 1.71	26.00	< 0.001	< 0.001	< 0.001
Give information	2.11 ± 0.76	2.25 ± 0.61	2.35 ± 0.51	6.74	0.034	0.182	0.157
Understand the patient’s perspective	1.89 ± 0.83	3.05 ± 0.64	3.00 ± 0.69	19.792	< 0.001	0.004	0.001
End the encounter	0.39 ± 0.58	0.89 ± 0.58	1.33 ± 0.48	22.136	< 0.001	0.037	< 0.001
SP Likert scale score	2.39 ± 0.61	3.50 ± 0.51	3.67 ± 0.68	28.35	< 0.001	< 0.001	< 0.001
Trainee Likert scale score	2.66 ± 0.84	3.28 ± 0.46	3.55 ± 0.78	13.81	0.001	0.037	0.008

*SP* Standardised patient

Participants expressed their perspectives on the use of SG in education, with 94.4% (17/18) indicating their enthusiasm for more opportunities to use this technology. 88.9% (16/18) acknowledged the value of SG in medical education. A majority (88.9%, 16/18) reported that awareness of being recorded did not affect their performance during interactions. Additionally, 88.9% (16/18) found that SG recordings provided feedback that was not available through traditional mounted wall cameras. Most residents believed that regular use of SG in teaching would enhance their verbal and nonverbal communication skills (Table 3).

**Table 3:** Survey on experience and education with smart glasses

Please let us know your opinion of using smart glasses (*n* = 18):	5 = strongly agree, 4 = agree, 3 = no opinion; 2 = disagree, and 1 = strongly disagree				
	5	4	3	2	1
I look forward to more opportunities to use smart glasses	77.8%	16.6%	5.6%	0%	0%
I see the value of using smart glasses for medical education	55.5%	33.3%	5.6%	5.6%	0%
I see the value of using smart glasses for nonverbal communication skill development	66.7%	22.2%	11.1%	0%	0%
I see the value of using smart glasses for verbal communication skill development	72.2%	22.2%	0%	5.6%	0%
The feedback I received from viewing the smart glasses was helpful	55.5%	27.8%	5.6%	11.1%	0%
I feel the smart glasses recording of me allowed an opportunity for additional feedback that did not exist	77.8%	11.1%	11.1%	0%	0%
Knowing the smart glasses were recording did not affect my performance during the encounter	50.0%	27.7%	5.6%	16.7%	0%

**Fig. 4:**
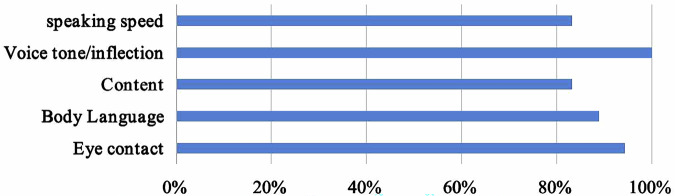
Additional feedback provided by smart glasses recordings during simulation training

Furthermore, participants were surveyed regarding the potential for additional feedback through SG recordings. Ninety-four percent (17/18) believed that SG allowed for enhanced feedback on eye contact, 88.9% (16/18) on body language, and all respondents agreed that it facilitated feedback on voice inflection and tone (Fig. 4). When discussing the limitations of using SG, 72.2% (13/18) noted that SG captured a limited viewing area, obscuring essential background information. Additionally, 94.4% (17/18) of residents reported difficulties and discomfort when viewing videos recorded with sudden or rapid head movements with the use of SG.

## Discussion

To the best of our knowledge, this is the first quasi-experimental study examining the efficacy of a simulation-based DPC curriculum augmented with SG, targeted at radiology residents in China. This paper delineates the incorporation of SG into clinical interactions as a feasible method for documenting residents’ performance from a first-person perspective. The findings of this novel programme shed light on substantial objective improvements in both SEGUE scores and self-assessed efficacy following the intervention. Notably, these enhancements have demonstrated enduring effects, with evidence of sustained benefits in residents’ practice 6 months post-training. Furthermore, our study demonstrated that using SG provides valuable feedback pertaining specifically to verbal and nonverbal communication skills.

According to the 2021 Chinese National Survey of Radiology Residency Training, such training programmes focus primarily on patient care and medical knowledge, with less emphasis on other essential competencies, such as DPC skills [3]. Results from a recent needs assessment survey of our training programme indicated that the availability of DPC training courses for radiology residents remains insufficient within Chinese institutions, and that most residents lack the confidence required to manage challenging conversations, despite generally positive attitudes towards DPC training [20]. Additionally, traditional curricula have tended to prioritise theoretical knowledge over the development of practical communication skills [3]. Prior to training, the baseline level of DPC competence among the radiology residents was relatively low, with an average SEGUE score of only 10.9 ± 2.9, and the satisfaction level of the SPs was similarly low at 2.4 ± 0.6 (Likert score), with both scores falling below half of the possible maximum. Therefore, an enhanced and effective DPC training programme, particularly addressing the concerns of radiology residents in China, should be considered a public health priority.

Simulation-based training has been increasingly used in medical education to improve communication skills across various subspecialties, including surgery [27] and paediatrics [28]. At its core, radiologists are placed in scenarios that range from routine to complex, necessitating effective communication with actors or patients [19, 29]. High-fidelity simulations represent a viable strategy for cultivating communication skills during radiology residency training. The implementation of such simulations facilitates both training and real-time performance assessment. The authenticity of these scenarios ensures they closely mimic real-life clinical practice [19, 29]. Several programmes have described the use of simulation-based approaches in DPC training [5, 22, 27, 28]. Our programme draws inspiration from a seminal work by Bai et al, which outlines a learner-centred, skills-focused, hands-on, multimodal surgical resident training programme [27]. In our study, the DPC training model was based on real-life hospital scenarios encompassing six distinct themes: error/apology, delivering bad news, cancelling examinations, handling challenging radiological reports, radiation risk counselling, and managing interactions with disgruntled referring physicians. Prior to each scenario, participants received brief didactic reviews of the respective topics, followed by group debriefing sessions that utilised videos from SG among the participants and the SP ensemble. These sessions emphasised the creation of a safe, non-judgemental environment. Post-training evaluations demonstrated a significant improvement in resident DPC competence (17.1 ± 3.7) and SP satisfaction (3.5 ± 0.5), with these improvements sustained for at least 6 months, aligning with findings from previous reports [5, 27].

In this study, the satisfaction levels of both SPs and radiology residents significantly increased following the training. In addition, there was a notable increase in consistency post-programme completion (intraclass correlation coefficient = 0.73, *p* = 0.005), indicating that the radiology residents’ evaluations of their communication competencies aligned more closely with those of the SPs after the programme compared with before. These results are in line with the findings of Bai et al, who reported that the consistency of the Likert between the SPs and surgical residents was 0.70 (*p* < 0.001) immediately following the programme [27]. In our study, SPs exhibited higher levels of satisfaction than radiology residents immediately after the programme, a trend that persisted 6 months later. This sustained satisfaction could be attributed to the programme’s emphasis on enhancing the residents’ verbal and nonverbal communication skills, which are often perceived as indicators of congruent communication that fosters effective doctor–patient relationships [11]. Indeed, effective gaze, empathy, and body orientation during DPC have been shown to be associated with higher patient satisfaction scores [30].

Learning is fundamental to feedback, and effective feedback is crucial in medical education [31]. Participating solely in isolated simulations is insufficient for significantly enhancing residents’ communication skills; a critical component of simulation-based training is the provision of debriefing and feedback following the simulation. During the post-simulation debriefing, the performance of the residents was scrutinised, and key teaching points were identified [32]. In 2017, DeBenedectis et al implemented a programme employing trained actors for both the training and assessment of communication skills through simulation [5]. This programme involved videotaping radiology residents and actors in simulations across six different scenarios. The initial videotaped simulations were debriefed by faculty and actors, with sessions repeated 2 weeks later to assess improvements. Residents received written feedback from both the faculty evaluator and the actor playing the patient for each scenario. In our study, we adopted a similar simulation-based training method and found comparable improvements. Our study, however, presents several advantages over that conducted by DeBenedectis et al, including the implementation of the ‘Ask-Tell-Ask’ model. This model, characterised by its simplicity and efficacy, facilitates bidirectional feedback that can significantly enhance physician clinical performance [5, 33]. Additionally, our training programme assessed the impact 6 months post-training, thereby increasing the power of our study. Another strength of our study was the utilisation of SG to record the performance of radiology residents during simulations. Recording simulations allows participants to review their performances subsequently and reflect on their strengths and areas for improvement [22]. SG, a widely used hands-free wearable device, offers unique first-person perspective feedback that is particularly valuable for evaluating body language, eye contact, and voice tone, unlike the traditional ‘birds-eye’ perspective from static cameras, which avoids causing distractions [15, 16, 34, 35]. In a study by Son et al, the use of SG by residents in an outpatient clinical setting resulted in improved patient satisfaction scores related to physician communication [36].

Of note, residents reported several disadvantages of using SG, including a limited recording view area. Occasionally, the SG video failed to capture the intended focus, and videos with sudden or rapid head movements would make it difficult for residents to review the recordings [37]. Further evaluation of egocentric video is necessary for assessing clinical DPC in simulations, particularly to address issues related to sudden head movements and misalignment of the camera with the participants’ line of sight.

This study has several limitations. First, although our radiology residency is moderately sized, it exhibited limited statistical power in longitudinal assessments, with only 18 residents completing these assessments. Our findings reflect experiences from a single, independent academic medical centre, which only provided initial insights and were insufficient to draw definitive conclusions. Future studies with larger, more diverse populations and advanced methodologies are necessary to validate these findings. This would strengthen the impact and applicability of the research outcomes. Second, the simulations employed SP evaluators, rather than actual patients. Third, our study lacked a control group. For instance, a comparative analysis between a “SG video group” and a control group without video or a group using a standard camera could elucidate the potential impact of SG videos when residents complete the pre- and post-self-assessment forms. Fourth, we did not evaluate the long-term effects of this programme, such as those manifesting 1 year post-training, although we did evaluate the effects 6 months after training.

In conclusion, this study introduced in detail the instructional methods utilised for clinical scenarios and effective feedback based on SG video to improve DPC skills, with an emphasis on both verbal and nonverbal competencies of radiology residents. This is the first study of its kind conducted in mainland China. Our findings indicate that this training programme effectively improves the DPC competencies of radiology residents, particularly in their verbal and nonverbal skills. Future studies involving multiple training programmes should be undertaken to further evaluate the efficacy and generalisability of this approach for radiology residents.

## Supplementary information

**Additional file 1: Supplemental Table 1.** Contents of Module 1. **Supplemental Table 2.** The post-session survey to assess attitudes towards the use of smart glasses. **Supplemental Table 3.** Data from the SEGUE and Likert scales before and after completion of the programme across different genders and resident grades. **Supplemental Table 4.** User manual of the smart glasses.

Supplementary video

## Data Availability

All data supporting the findings of this study are available within the paper and its Supplementary material files. The results of the pre-course training needs survey have been published in our previous research work under the identifier https://bmcresnotes.biomedcentral.com/articles/10.1186/s13104-024-06779-8.

## Abbreviations

DPC: Doctor-patient communication
SG: Smart glasses
SP: Standardised patient
