# Supplementary material for: Efficacy of a smart glass-enhanced training programme for core doctor-patient communication skills among radiology residents in China
Source: Eur Radiol Exp. 2025 Sep 19;9:92. doi: 10.1186/s41747-025-00630-w (PMC12449284; doi:10.1186/s41747-025-00630-w)
Supplement: Supplementary file 1 — Additional file 1: Supplemental Table 1. Contents of Module 1. Supplemental Table 2. The post-session survey to assess attitudes towards the use of smart glasses. Supplemental Table 3. Data from the SEGUE and Likert scales before and after completion of the programme across different genders and resident grades. Supplemental Table 4. User manual of the smart glasses. [file 41747_2025_630_MOESM1_ESM.pdf]

Efficacy of a smart glass-enhanced training program for core doctor-patient communication skills among radiology residents in China

ELECTRONIC SUPPLEMENTARY MATERIAL

Eur Radiol Exp (2025) Xiao Y, Lian G, Zhang J, et al.

Supplemental Table 1. Contents of Module 1

Theme	Content	Duration
Ethical and professional principles of DPC	• Learn ethics: understand key ethical principles like respect for patients and fairness.<br>• Know Standards: familiarize with professional guidelines for DPC.<br>• Make good decisions: develop skills to make ethical choices in challenging situations.<br>• Build trust: emphasize the importance of trust and respect in patient relationships.	1 h
Effective verbal and nonverbal skills for DPC	• Improve communication: practice clear and empathetic verbal communication.<br>• Understand body language: learn about non-verbal cues like tone, eye contact, and posture.<br>• Build rapport: develop skills to connect with patients and make them feel valued.<br>• Adapt communication: adjust communication styles to meet different patient needs.	1 h
DPC skills in radiology from a nursing perspective	• Apply communication techniques in real-world scenarios: equip students with practical verbal and non-verbal communication strategies that can be applied in common radiology situations, such as explaining procedures, addressing patient concerns, and providing reassurance during imaging processes.<br>• Promote teamwork: engage students in activities that highlight the importance of communication among healthcare professionals, showing how teamwork improves patient care in radiology.<br>• Assess communication skills: encourage students to evaluate their communication effectiveness through peer feedback and reflection, promoting ongoing improvement in their patient interactions.	1 h

DPC Doctor-patient communication.

Supplemental Table 2. The post-session survey to assess attitudes towards the use of smart glasses

Name:		
Year of residency:		
Date:		
Please let us know your opinion of using smart glasses in this training programme:	Yes	No
1. I look forward to more opportunities to use smart glasses	☐	☐
2. I see the value of using smart glasses for medical	☐	☐
education	☐	☐
3. I see the value of using smart glasses for nonverbal		
communication skill development	☐	☐
4. I see the value of using smart glasses for verbal		
communication skill development	☐	☐
5. The feedback I received from viewing the smart glasses		
was helpful	☐	☐
6. Knowing the smart glasses were recording did not affect		
my performance during the encounter	☐	☐
7. I feel the smart glasses recording of me allowed an		
opportunity for additional feedback that did not exist		
8. Additional feedback provided by smart glasses include:	☐	☐
• Eye contact	☐	☐
• Body language	☐	☐
• Voice inflection/tone	☐	☐
• Content	☐	☐
• Speaking speed	☐	☐
9. Smart glasses obscure important background information		
by capturing a limited field of view	☐	☐
10. Does watching recorded video with smart glasses make you feel uncomfortable?		

Supplemental Table 3. Data from the SEGUE and Likert scales before and after completion of the programme across different genders and resident grades

Variable	Female	Male	*p*-value	Year-2 residents	Year-3 residents	*p*-value
SEGUE total score						
Pretest	10.67 ± 2.23	11.22 ± 3.52	0.789	10.11 ± 2.61	11.78 ± 3.03	0.246
Post-test	16.89 ± 3.29	17.22 ± 4.21	0.824	14.33 ± 2.50	19.78 ± 2.38	**0.002**
6 months post	17.33 ± 2.87	18.11 ± 3.48	0.503	15.89 ± 2.57	19.56 ± 2.35	**0.011**
Set the stage						
Pretest	2.55 ± 0.53	2.00 ± 1.32	0.349	2.11 ± 0.78	2.44 ± 1.23	0.303
Post-test	4.00 ± 1.00	4.01 ± 0.98	1.000	3.11 ± 0.33	4.89 ± 0.33	**< 0.001**
6 months post	4.11 ± 0.78	4.22 ± 0.97	0.705	3.56 ± 0.73	4.78 ± 0.44	**0.002**
Elicit information						
Pretest	4.00 ± 0.86	4.55 ± 0.72	0.242	4.00 ± 0.71	4.55 ± 0.88	0.156
Post-test	6.44 ± 1.67	6.67 ± 1.94	0.687	5.33 ± 1.41	7.78 ± 1.09	**0.002**
6 months post	6.66 ± 1.58	6.66 ± 1.90	0.823	5.56 ± 1.50	7.09 ± 0.41	**0.005**
Give information						
Pretest	2.00 ± 0.50	2.22 ± 0.97	0.242	2.22 ± 0.44	2.00 ± 1.00	0.799
Post-test	2.44 ± 0.53	2.67 ± 0.71	0.518	2.23 ± 0.41	2.89 ± 0.60	**0.020**
6 months post	2.44 ± 0.53	2.44 ± 0.52	0.357	2.33 ± 0.50	2.78 ± 0.44	0.065

Understand the patient's perspective						
Pretest	1.78 ± 0.44	2.00 ± 1.11	0.417	2.22 ± 0.67	1.56 ± 0.88	0.095
Post-test	3.00 ± 0.50	3.11 ± 0.78	0.685	2.89 ± 0.60	3.22 ± 0.67	0.265
6 months post	2.67 ± 0.50	3.33 ± 0.70	**0.039**	3.00 ± 0.70	3.00 ± 0.71	1.000
End the encounter						
Pretest	0.33 ± 0.50	0.44 ± 0.52	0.638	0.56 ± 0.52	0.22 ± 0.44	0.159
Post-test	1.00 ± 0.50	0.78 ± 0.66	0.396	0.77 ± 0.67	1.00 ± 0.50	0.396
6 months post	1.22 ± 0.44	1.44 ± 0.53	0.331	1.44 ± 0.53	1.22 ± 0.44	0.331
SP Likert scale score						
Pretest	2.33 ± 0.50	2.44 ± 0.72	0.551	2.44 ± 0.72	2.33 ± 0.50	0.551
Post-test	3.44 ± 0.52	3.55 ± 0.53	0.647	3.22 ± 0.44	3.78 ± 0.44	0.609
6 months post	3.67 ± 0.87	3.66 ± 0.50	0.771	3.56 ± 0.72	3.78 ± 0.67	0.437
Trainee Likert scale score						
Pretest	2.78 ± 0.83	2.55 ± 0.88	0.494	2.44 ± 0.72	2.89 ± 0.92	0.204
Post-test	3.33 ± 0.50	3.22 ± 0.44	0.609	3.33 ± 0.50	3.22 ± 0.44	0.609
6 months post	2.44 ± 0.53	2.67 ± 0.71	0.577	3.78 ± 0.83	3.33 ± 0.71	0.187

SP Standardized patient

Supplemental Table 4 User manual of the smart glasses

**Product name**: Eyeglass Camera
**Model number**: MJSV01FC
**Internal storage**: 32GB*
**Processor**: Qualcomm Snapdragon Platform
**Camera parameters**:
• Telephoto Camera 8MP 5X (120mm)
• Ultra-clear Wide-angle Camera 50MP (28mm)
**Display**: Sony Micro OLED, 0.23 inches
**Bluetooth**: Bluetooth 5.0
**Wireless network**: 802.11 a/b/g/n/ac, supporting Wi-Fi 2.4GHz	Wi-Fi 5GHz
**Rated input**: 5V, 2A
**Lithium polymer battery**: 1020mAh / 3.95Wh (typical)
**Product weight**: 100g (excluding quick-release frame)
**Frame size**: 54-19-147(Lens Width, Bridge Width, Temple Length)
**Operating temperature**: from -10 ℃ to ~ 35 ℃
